# Survival and Morphological Changes of *Clostridium butyricum* Spores Co-Exposed to Antibiotics and Simulated Gastrointestinal Fluids: Implications for Antibiotic Stewardship

**DOI:** 10.3390/microorganisms13061347

**Published:** 2025-06-10

**Authors:** Yi-Meng Yang, Meng-Yue Zhang, Ying-Ying Wu, Lu Zhang, Yi-Xuan Zhang

**Affiliations:** 1 School of Life Science and Biopharmaceutics, Shenyang Pharmaceutical University, Shenyang 110016, China; 2 Hangzhou Grand Biologic Pharmaceutical Inc., Hangzhou 310030, China

**Keywords:** *Clostridium butyricum*, probiotic spores, antibiotic tolerance, gastrointestinal simulation, scanning electron microscopy, co-administration

## Abstract

Probiotics are often advised to be taken separately from antibiotics due to their sensitivity to antibiotic activity and gastrointestinal (GI) stress. However, *Clostridium butyricum* spores, as next-generation probiotics, may withstand concurrent use with antibiotics due to their unique structural adaptations. This study systematically evaluated the survival rates and morphological changes of *C. butyricum* spores exposed to 10 clinically relevant antibiotics in simulated gastric/intestinal fluids, exploring their feasibility for co-administration with antibiotics. Survival rates of *C. butyricum* spores were tested against 10 antibiotics across four classes (β-lactams, macrolides, aminoglycosides, and tetracyclines) in simulated GI fluids. Time–kill curves analyzed spore survival over 0–4 h, while scanning electron microscopy (SEM) observed spore wall integrity and morphological changes under different conditions. The spore survival rates remained >89% in intestinal fluid and >60% in gastric fluid across all antibiotics. SEM revealed gastric acid and proteolysis increased spore wall fragmentation, reducing resistance, whereas the intestinal environment preserved spore integrity. This study was the first to demonstrate that *C. butyricum* spores can survive simultaneous exposure to antibiotics in the gastrointestinal tract, challenging traditional probiotic usage guidelines. The findings support their co-administration with antibiotics to simplify dosing regimens and improve medication adherence. Such an approach advances antimicrobial stewardship by optimizing therapeutic strategies for antibiotic–probiotic combinations.

## 1. Introduction

The human gut microbiome plays a pivotal role in maintaining host health, orchestrating nutrient metabolism, immune modulation, and pathogen resistance [1]. However, its equilibrium is frequently disrupted by antibiotic therapies, which indiscriminately deplete commensal microbes and promote dysbiosis-associated pathologies such as opportunistic infections and chronic inflammation [2,3]. While probiotics are widely recommended to mitigate antibiotic-associated dysbiosis, their efficacy is often compromised by poor survival in the harsh gastrointestinal environment and susceptibility to antibiotic activity [4]. Conventional probiotics, such as *Lactobacillus* and *Bifidobacterium*, require temporal separation from antibiotics to avoid inactivation [5]. This practice complicates dosing regimens and reduces patient adherence, particularly in vulnerable populations. This complexity not only undermines treatment outcomes but also jeopardizes antimicrobial stewardship efforts, as non-compliance with dosing schedules may lead to incomplete antibiotic courses and promote resistance [6,7].

*Clostridium butyricum* was first isolated from pig intestines by Prazmowski in 1880. Since then, its presence has been identified in diverse environments [8]. Currently, some strains of *C. butyricum* have become widely used probiotic products in Asia and Europe [9]. *C. butyricum* is a spore-forming, Gram-positive, strictly anaerobic bacterium. It is typically one of the first probiotics to colonize the infant gut and is detected in 10% to 20% of adult intestines [10]. Studies have shown that *C. butyricum* can ferment undigested dietary fiber in the animal intestine and produce short-chain fatty acids, mainly butyric acid [11]. Butyrate is the primary energy source for colonic epithelial cells and can enhance intestinal barrier function by regulating the production of mucin [12]. Additionally, *C. butyricum* promotes the production of anti-inflammatory lipid metabolites in mouse colon tissue. These lipid metabolites help colonic T cells secrete anti-inflammatory IL-10, enhancing the immune regulation of the host intestine [10]. Experiments have demonstrated that *C. butyricum* can alleviate inflammatory bowel disease by targeting intestinal macrophages to modulate immunity [13,14], and improve clinical symptoms related to *Clostridium difficile* infection through enhancing innate antibacterial immunity and cytokine-mediated immune responses [15]. Recent studies have found that apart from gastrointestinal disorders, *C. butyricum* also exhibits beneficial effects in enhancing human resistance against influenza virus pneumonia [16], improving cognitive impairments in obese patients [17], and treating *Escherichia coli*-induced endometritis [18].

Additionally, *C. butyricum* possesses a unique stratified spore structure, exhibiting structural adaptations (e.g., multilayered outer coat, dipicolinic acid-rich core) that confer resistance to heat, acid, and enzymatic degradation [19,20]. These adaptations enable the spores to survive gastric transit, colonize the intestine, and germinate into metabolically active cells. Despite these advantages, clinical guidelines for *C. butyricum* preparations remain empirically based, requiring temporal separation from antibiotics. Overcoming this conservative approach necessitates systematic studies evaluating spore survival under combined antibiotic and digestive-fluid challenges, despite emerging evidence suggesting their potential for co-administration [21].

Due to the structural advantages of *Clostridium butyricum* spores, we believe that the spores of *C. butyricum* RH_2_ have the ability to resist certain antibiotics and can be clinically used in combination. Therefore, in this experiment the experimental methods were based on clinical practical applications. By studying the tolerance of *C. butyricum* spores to 10 clinically common antibiotics in simulated gastric (pH 3.5) and intestinal (pH 6.8) fluids, this study fills this knowledge gap, challenges the existing administration model of *C. butyricum*, and provides a scientific basis for optimizing the combined administration strategy of *C. butyricum* and antibiotics.

## 2. Materials and Methods

### 2.1. Bacterial Strain

*C. butyricum* RH_2_ spore powder (2.0 × 10^10^ CFU/g) was provided by Hangzhou Grand Biologic Pharmaceutical Inc. (Zhejiang, China).

### 2.2. Simulated Digestive Fluids

Simulated gastric fluid (SGF): Prepared with 2.0 g/L NaCl and 3.2 g/L pepsin (Beijing Solarbio, ≥2500 U/mg), the solution was adjusted to pH 3.5 ± 0.1 using HCl and then sterile-filtered (0.22 μm, Biosharp, Labgic Technology Co., Ltd., Hefei City, China). The pH was set to 3.5 to simulate the typical postprandial human gastric acid environment, which usually has a pH around 3.5. This pH value is particularly relevant for probiotics, which are typically administered postprandially to maximize their survival and efficacy.

Simulated intestinal fluid (SIF): Composed of 6.8 g/L KH_2_PO_4_ and 10 g/L pancreatin (USP-grade, Beijing BOAOtoda Technology Co., Ltd., Beijing, China), the solution was adjusted to pH 6.8 ± 0.1 using NaOH/HCl and then sterile-filtered (0.22 μm). The pH was set to 6.8 to simulate the typical postprandial intestinal environment, as the small intestine generally has a pH ranging from 6.5 to 7.5 after a meal. This pH range is particularly relevant for probiotics, which are typically administered postprandially to maximize their survival and efficacy.

### 2.3. Medium and Antibiotic Preparation

Trypticase peptone yeast extract (TYP) agar medium: 10 g of peptone, 0.3 g of L-cysteine, 10 g of tryptone, 2.5 g of dipotassium hydrogen phosphate, 5 g of soy peptone, 3 g of sodium chloride, 3 g of yeast powder, 0.3 g of thioglycolic acid, 10 g of glucose, and 15 g of agar powder were dissolved in purified water to make up to 1000 mL. The pH was adjusted to between 6.3 and 6.7, and the medium was sterilized by high-pressure sterilization at 115 °C for 20 min.

Ten antibiotics were selected based on clinical prevalence (Table 1). The maximum single dose of each antibiotic was determined based on the drug instructions. Considering the average volumes of the human stomach and intestines, the maximum concentration of each antibiotic was calculated for in vitro experiments (see Table 1). It should be noted that this concentration represents a specific experimental concentration chosen for the purpose of this study and does not necessarily reflect the actual clinical dosing levels.

### 2.4. Determination of Spore Survival Rate

*C. butyricum* RH_2_ spores were dispersed in a diluent (0.5% yeast extract, 1% Tween 80, 0.025% L-cysteine; pH 6.5 ± 0.2) and vortexed at 200 rpm for 15 min to achieve a homogeneous suspension (2.0 × 10^7^ CFU/mL). Spore suspensions (1 mL) were mixed with 10 mL of SGF/SIF containing antibiotics (Table 1) and incubated at 37 °C (50 rpm). Aliquots (1 mL) were collected at 0, 1, 2, 3, and 4 h, centrifuged (10,000× *g*, 5 min), and resuspended in diluent. Serial dilutions were then plated on trypticase peptone yeast extract (TPY) agar medium. The plates were incubated anaerobically at 37 °C for 48 h, and colonies were counted (threshold of 30–300 CFU/plate), to reflect the survival status of spores. Survival rates (%) were calculated using the following formula, allowing for the generation of time–kill curves with time on the x-axis and the number of viable spores or the survival rate of viable spores on the y-axis:

Survival rate (%) = Ai/Ai′ × 100%

In this formula, Ai (experimental group) denotes the viable cell count (CFU/mL) of the strain after i hours of co-incubation with antibiotics in SGF or SIF. Ai′ (control group) denotes the viable cell count (CFU/mL) of the strain after i hours of incubation in SGF or SIF without antibiotics.

### 2.5. Scanning Electron Microscopy (SEM)

Spores were fixed in 2.5% glutaraldehyde, dehydrated via ethanol gradient, critical-point dried, and sputter-coated with gold. Morphology was analyzed using a Hitachi SU8010 SEM at 12,000× and 65,000× magnifications.

### 2.6. Statistical Analysis

Data are expressed as mean ± SD (*n* = 3). One-way ANOVA with Tukey’s post-hoc test was performed using GraphPad Prism 10.0 (* *p* < 0.05, ** *p* < 0.01, *** *p* < 0.001).

## 3. Results

### 3.1. Survival in Simulated Gastric Fluid (SGF)

When *C. butyricum* spores were co-incubated with antibiotics in simulated gastric fluid (SGF), significant differences in spore survival rates were observed across time points and antibiotic classes (Table 2), accompanied by distinct morphological changes. Specific colony counts (Table S1) and corresponding bar charts (Figure S1) are provided in the Supplementary Materials.

**Figure 1:**
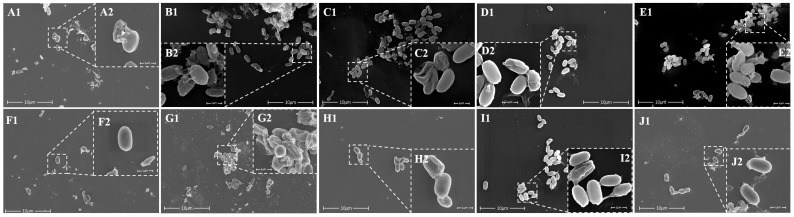
Scanning electron microscopy images of *Clostridium Butyricum* spores in SGF containing β-lactam antibiotics. (**A**) Cefalexin (**B**) cefradine (**C**) cefuroxime (**D**) ampicillin (**E**) amoxicillin; macrolide antibiotic (**F**) azithromycin (**G**) roxithromycin; aminoglycoside antibiotic (**H**) gentamicin; tetracycline antibiotic (**I**) tetracycline (**J**) doxycycline. (In each figure, the magnification of 1 is 12,000× times, and the magnification of 2 is 65,000× times.).

(1) β-Lactam antibiotics: *C. butyricum* spores exhibited moderate tolerance to the combined stress of SGF and β-lactam antibiotics. In cefalexin, cefuroxime, and amoxicillin, the spore survival rates declined steadily within the first 2–3 h before stabilizing, with final 4 h survival rates of 68.18 ± 8.68%, 83.14 ± 4.75%, and 60.33 ± 11.52%, respectively. In cephradine, spore survival rates remained unaffected within 2 h but decreased rapidly between 2 and 3 h, achieving a final 4 h survival rate of 73.48 ± 8.35%. Ampicillin had minimal impact on survival rates, with a 4 h survival rate of 92.99 ± 13.83%, indicating that *C. butyricum* RH_2_ spores showed higher tolerance to ampicillin than to amoxicillin. Scanning electron microscopy (SEM) revealed that spores exposed to ampicillin displayed no wrinkling or damage, whereas other β-lactam antibiotics induced structural defects in RH_2_ spores, such as folding, perforation, and spore wall rupture (Figure 1A–E).
(2) Macrolide antibiotics: After 4 h of co-incubation with macrolide drugs in SGF, azithromycin had minimal impact on RH_2_ spore survival rates (93.28 ± 5.85%), whereas spore survival rates in roxithromycin decreased gradually, with a final 4 h survival rate of 67.68 ± 12.97%. SEM images showed that RH_2_ spores exposed to azithromycin remained relatively intact, despite minor cellular debris and sporadic perforations. In contrast, spore damage was more severe in roxithromycin, characterized by pronounced outer wall folding and extensive perforations (Figure 1F,G).
(3) Aminoglycoside antibiotics: Following 4 h co-incubation with gentamicin in SGF, *C. butyricum* spore viability reached 95.98 ± 12.08%, indicating minimal impact. SEM confirmed spore integrity under these conditions, with only slight folding observed in a small subset of spores (Figure 1H).
(4) Tetracycline antibiotics Co-incubation with tetracycline in SGF for 4 h resulted in a survival rate of 96.10 ± 13.74%, with negligible effect on spore viability. However, tolerance was moderately reduced in doxycycline, with viability declining gradually to a final 4 h survival rate of 73.57 ± 3.44%. SEM showed that tetracycline-exposed spores retained intact morphology with occasional minor folding, while doxycycline-exposed spores exhibited cell wall wrinkling, structural damage, and cytoplasmic leakage (Figure 1I,J).

### 3.2. Survival in Artificial Intestinal Fluid (SIF)

When RH_2_ spores were co-incubated with antibiotics in simulated intestinal fluid (SIF), the spores exhibited strong tolerance to all tested antibiotics across different time points. Survival rates fluctuated within a reasonable margin of error and did not show any significant decline, maintaining a survival rate of ≥89% after 4 h of co-incubation (Table 3), which highlights their robust stress resistance. Specific colony counts (Table S2) and corresponding bar charts (Figure S2) are provided in the Supplementary Materials. Scanning electron microscopy (SEM) also showed that spore morphology was generally well-preserved with minimal differences under various antibiotic treatments.

**Figure 2:**
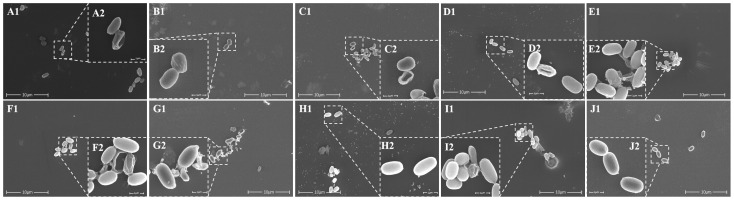
Scanning electron microscopy images of *Clostridium butyricum* spores in SIF containing β-lactam antibiotics. (**A**) Cefalexin (**B**) cefradine (**C**) cefuroxime (**D**) ampicillin (**E**) amoxicillin; macrolide antibiotic (**F**) azithromycin (**G**) roxithromycin; aminoglycoside antibiotic (**H**) gentamicin; tetracycline antibiotic (**I**) tetracycline (**J**) doxycycline. (In each figure, the magnification of 1 is 12,000× times, and the magnification of 2 is 65,000× times.).

(1) β-Lactam antibiotics: Compared to SGF, C. butyricum spores exhibited higher tolerance to penicillins in SIF, with survival rates of 90.91 ± 7.88% for ampicillin and 97.86 ± 3.27% for amoxicillin. Cephalosporin antibiotics such as cefalexin (89.51 ± 5.35%), cephradine (96.5 ± 10.64%), and cefuroxime (89.81 ± 9.4%) also showed high tolerance. SEM observations revealed that spores remained predominantly smooth and intact, with minimal cellular fragmentation in the background, despite occasional minor folding or perforations (Figure 2A–E).
(2) Macrolide antibiotics: Co-incubation with azithromycin (92.42 ± 13.12%) and erythromycin (95.16 ± 6.44%) in SIF preserved spore integrity. SEM detected no significant structural damage, except for localized wrinkling in a subpopulation of azithromycin-treated spores (Figure 2F,G).
(3) Aminoglycoside antibiotics: C. butyricum spores showed a high survival rate (97.81 ± 19.17%), with SEM confirming that most spores maintained smooth and intact morphology (Figure 2H).
(4) Tetracycline antibiotics: Spore viability remained high in tetracycline (93.87 ± 9.53%) and doxycycline (91.36 ± 31.31%). SEM images showed that most spores had intact, smooth surfaces with minimal debris (Figure 2I,J).

## 4. Discussion

*Clostridium butyricum*, first isolated in 1880, is a spore-forming probiotic detected in 10–20% of adult intestines. Widely used in Asia/Europe, it ferments dietary fiber into butyrate, a key energy source for colonocytes which strengthens the intestinal barrier via mucin regulation [11,12]. It also promotes anti-inflammatory lipid metabolites, enhancing IL-10 secretion and immune regulation [10]. Studies show that it alleviates colitis, improves *C. difficile* infection symptoms, and boosts antiviral immunity [15,16]. Emerging benefits include cognitive improvement in obesity and treatment of endometritis [17,18].

Previous studies have explored the tolerance of *C. butyricum* to adverse environmental conditions. However, this current research distinguished itself in two key aspects: Firstly, it focused on the viable spores of *C. butyricum* RH_2_. This probiotic strain has been marketed in China since 2004, yet surprisingly few investigations have been conducted on its spore-form’s antibiotic resistance. Secondly, some studies on spore tolerance to antibiotics have been limited to aqueous solution environments, failing to simultaneously consider the dual pressures of the human gastrointestinal tract. For example, Mitsuboshi et al. [22] evaluated the Japanese strain *C. butyricum* Miya-BM by suspending it with antibiotics in water at 55 °C for 10–360 min, then assessing growth inhibition via plate culture, without considering the combined effects of gastric/intestinal fluids and antibiotics on spore viability. This study broke through this limitation by investigating the survival of *C. butyricum* RH_2_ spores under dual stress conditions: simultaneous exposure to antibiotics and simulated gastrointestinal environments. To enhance clinical relevance, antibiotic prescription data from primary healthcare units in China were analyzed to select 10 frequently used antibiotics across major classes (e.g., β-lactams, aminoglycosides, tetracyclines, macrolides) for testing. The experimental design focused on examining spore survival rates under the combined pressures of these 10 common antibiotics and either gastric or intestinal fluids. By integrating real-world antibiotic usage patterns with physiological gastrointestinal (GI) conditions, this approach more accurately mimicked in vivo scenarios, addressing the gap in the understanding of spore tolerance within actual digestive tract environments. At the same time, through scanning electron microscopy, it revealed that RH_2_ spores maintained significant structural integrity under these combined stresses, providing direct morphological evidence for the feasibility of co-administering *C. butyricum* with antibiotics and offering a new perspective for optimizing probiotic–antibiotic combination strategies.

This study systematically evaluated the resilience of *C. butyricum* spores under dual stress from antibiotics and simulated gastrointestinal fluids, providing critical evidence to challenge the traditional paradigm of temporal separation between probiotics and antibiotics. Our findings showed that *C. butyricum* spores exhibited exceptional tolerance to 10 clinically relevant antibiotics in simulated intestinal fluid (SIF), maintaining ≥89% survival after 4 h (Table 3). While no identical studies exist, analogous research by Zhang et al. [23] and Li et al. [24] demonstrated that *C. butyricum* can tolerate high temperatures and broad pH ranges, with antibiotic resistance varying by strain. However, these studies did not investigate combined stress effects of environmental stress on spore resistance to antibiotic damage.

Relatively, in simulated gastric fluid, the survival rate of *C. butyricum* spores (≥60%) after 4 h co-incubation with different antibiotics was lower than that in simulated intestinal fluid (≥89%). Scanning electron microscopy (SEM) observations further supported this finding, revealing more fragmented spores and worse structural integrity in SGF (Figure 1 and Figure 2). This discrepancy likely arose from the synergistic effects among gastric acidity, pepsin activity, and antibiotics. The acidity of SGF (pH 3.5 in this study) is inherently lower than that of SIF (pH 6.8), and the hydrolytic effects of pepsin on spore cell walls and membranes in SGF may further compromise survival. Additionally, the 4 h co-incubation time used in this study represented a relatively prolonged retention period in the stomach. In real-life scenarios, drugs and probiotics typically empty from the stomach within 1–4 h, during which time *C. butyricum* spore survival would likely be higher. Kheadr et al. [25] similarly reported that environmental stresses (e.g., acid, bile salts, H_2_O_2_) modulated antibiotic sensitivity and survival rates in probiotics (*Bifidobacterium*), with significant strain-specific variations. This underscores the need to consider stress–antibiotic interactions in probiotic–antibiotic co-therapy. Collectively, *C. butyricum* spores maintained ≥60% survival against β-lactams, macrolides, aminoglycosides, and tetracyclines, and this superior tolerance compared to vegetative cells of conventional probiotics can likely be attributed to the unique spore structure of *C. butyricum*.

The robust tolerance of *C. butyricum* spores likely stems from their structural adaptations, including multi-layered protein coats and dipicolinic acid-rich cores. A unique exosporium layer restricts antibiotic diffusion, conferring resistance to enzymatic degradation and antibiotic penetration [20]. Even under moderate gastric conditions (pH 3.5) with concurrent antibiotic stress, spores maintained >60% viability, a feat unattainable by non-spore-forming probiotics. Clinical evidence also supports this finding, such as *C. butyricum* supplementation reducing the incidence of antibiotic-associated diarrhea (AAD) in patients [26]. Additionally, in lung cancer treatment, *C. butyricum* demonstrates synergistic therapeutic effects when co-administered with chemotherapeutics, highlighting its translational potential [27]. This contrasts sharply with traditional probiotics like lactic acid bacteria (LAB) and *Bifidobacterium*, which lack such protective mechanisms [28]. For example, most LAB are sensitive to exposed antibiotics [29], while many *Bifidobacterium* strains require stress-resistant selection due to acid intolerance [30], let alone survival in harsh gastric environments combined with antibiotics. Moreover, antibiotic-resistant traditional probiotics pose risks of resistance gene transfer, a concern absent in *C. butyricum*.

Current clinical guidelines recommend separating probiotics from antibiotics by 2–3 h to avoid microbial inactivation. However, our data suggest *C. butyricum* spores may bypass this restriction, maintaining ≥89% survival against 10 antibiotics in simulated intestinal fluid (Table 3) and ≥60% in simulated gastric fluid (Table 2), implying potential feasibility for concurrent use. Critically, simplifying dosing regimens through co-administration aligns with antimicrobial stewardship goals by improving patient adherence and safety, reducing unnecessary antibiotic discontinuation, a key factor in curbing resistance development [6,7]. This targeted approach addresses adherence challenges in pediatric and geriatric populations by streamlining treatment protocols.

Although *C. butyricum* exhibits numerous advantages over other probiotics, there are still some limitations to its use. Studies have shown that certain *C. butyricum* strains can cause botulism in infants, and necrotizing enterocolitis in preterm neonates during administration, which may be related to the toxin proteins encoded by the *Clostridium* toxin-related genes in the tyrosine-producing *Clostridium* or the production of substances related to *Clostridium* toxins, such as botulinum neurotoxins A, B, E, and F, or *Clostridium* perfringens toxins α, β, and ε. These toxins can cause the aforementioned diseases in infants [8,31]. Meanwhile, some non-toxigenic tyrosine-producing *Clostridium* strains may trigger an inflammatory cascade reaction in the intestines of preterm infants with underdeveloped digestive systems by utilizing undigested lactose to produce excessive amounts of butyric acid, exacerbating the burden on the intestinal barrier and causing necrotizing enterocolitis in preterm neonates [32]. Additionally, there are cases of related bacteremia occurring after treatment with *C. butyricum* probiotics. The pathogenic mechanism has not yet been elucidated, and it is speculated that this may be related to the patient suffering from multiple comorbidities (including immunosuppressive treatment and intraabdominal problems) simultaneously [31]. This study used *C. butyricum* RH_2_, a microecological preparation drug launched in China in 2004, which has not had any toxicity-related incidents in over 20 years, proving the safety of this strain. However, it is still recommended to pay attention to the age and pathological conditions of patients when using *C. butyricum* RH_2_ clinically, and avoid using it on preterm infants.

While this study provides foundational in vitro evidence, dynamic validation in human gastrointestinal tracts remains necessary. For example, postprandial gastric pH fluctuations (0.9–7.0) and peristalsis influence *C. butyricum* spore transit time. Future work should explore spore survival across pH gradients simulating fasting and fed states, as proposed by recent gut-on-a-chip models [33]. Additionally, expanding antibiotic coverage to quinolones and nitroimidazoles would refine clinical guidance, especially given rising resistance to these antibiotic classes [34,35]. Tracking in vivo spore germination and colonization during antibiotic therapy is also critical to confirm functionality and benefits [21], which will require substantial follow-up studies.

## 5. Conclusions

This study establishes *C. butyricum* spores as a paradigm-shifting probiotic capable of withstanding antibiotic–gastrointestinal challenges that incapacitate conventional strains. By enabling potential co-administration with antibiotics, this organism could revolutionize clinical practice. It provides a practical strategy for antimicrobial stewardship by minimizing dosing complexity, thereby enhancing treatment adherence and reducing the risk of incomplete antibiotic courses, a key driver of resistance. However, varying survival responses of other probiotics to dual antibiotic–gastrointestinal stress necessitate tailored dosing strategies, underscoring the importance of strain–antibiotic compatibility assessments in therapeutic protocols.

## Figures and Tables

**Table 1:** Concentrations antibiotics in SGF and SIF.

Class	Antibiotics	Concentration in SGF (μg/mL)	Concentration in SIF (μg/mL)	Manufacturer	
β-lactams	Cephalexin	333.3	250	Sinopharm Shantou Jinshi Pharmaceutical Co., Ltd., Shantou, China	
	Cefradine	333.3	250	Shandong Lukang Pharmaceutical Co., Ltd., Jining, China	
	Cefuroxime	333.3	250	Sinopharm Zhijun (Shenzhen) Pharmaceutical Co., Ltd., Shenzhen, China	
	Ampicillin	500	375	Zhuhai United Laboratories (Zhongshan) Co., Ltd., China	
	Amoxicillin	333.3	250	CSPC Zhongnuo Pharmaceutical (Shijiazhuang) Co., Ltd., Shijiazhuang China	
Macrolides	Azithromycin	333.3	250	Beijing Sihuan Pharmaceutical Co., Ltd., Beijing, China	
	Roxithromycin	200	150	Shanxi Tongda Pharmaceutical Co., Ltd., Datong, China	
Aminoglycosides	Gentamicin	106.7	80	Hunan Qianjin Xiangjiang Pharmaceutical Co., Ltd., Zhuzhou, China	
Tetracyclines	Tetracycline	333.3	250	Guangdong Huanan Pharmaceutical Group Co., Ltd., Dongguan, China	
	Doxycycline	133.3	100	Hunan Xiangya Pharmaceutical Co., Ltd., Changsha, China	

SGF = simulated gastric fluid; SIF = simulated intestinal fluid.

**Table 2:** Survival rate of *Clostridium butyricum* spores in SGF containing antibiotics.

Sampling Time (h)			0	1	2	3	4
**Average Survival Rate(%)**	β-lactams	Cephalexin	100.00 ± 0.00	84.00 ± 7.64	74.70 ± 12.49 *	67.69 ± 8.42 **	68.18 ± 8.68 **
		Cefradine	100.00 ± 0.00	95.18 ± 3.90	98.81 ± 9.58	76.35 ± 8.19 *	73.48 ± 8.35 **
		Cefuroxime	100.00 ± 0.00	90.97 ± 6.76	80.24 ± 6.74	82.10 ± 18.17	83.14 ± 4.75
		Ampicillin	100.00 ± 0.00	98.18 ± 12.58	95.55 ± 9.91	93.10 ± 5.47	92.99 ± 13.83
		Amoxicillin	100.00 ± 0.00	84.00 ± 3.78	61.66 ± 4.11 ***	63.93 ± 5.36 ***	60.33 ± 11.52 ***
	Macrolides	Azithromycin	100.00 ± 0.00	100.99 ± 2.97	98.63 ± 14.12	93.27 ± 10.80	93.28 ± 5.85
		Roxithromycin	100.00 ± 0.00	95.11 ± 11.21	79.00 ± 12.12	75.46 ± 16.74	67.68 ± 12.97 *
	Aminoglycosides	Gentamicin	100.00 ± 0.00	94.32 ± 14.22	96.44 ± 6.22	94.28 ± 7.30	95.98 ± 12.08
	Tetracyclines	Tetracycline	100.00 ± 0.00	98.18 ± 6.64	92.89 ± 3.81	98.31 ± 8.46 *	96.10 ± 13.74
		Doxycycline	100.00 ± 0.00	85.93 ± 7.01 *	81.33 ± 1.53 **	80.30 ± 8.04 **	73.57 ± 3.44 ***

SGF = simulated gastric fluid. Average survival rate at 1 h, 2 h, 3 h, 4 h vs. 0 h; * *p* < 0.05, ** *p* < 0.01, *** *p* < 0.001.

**Table 3:** Survival rate of *Clostridium butyricum* spores in SIF containing antibiotics.

Sampling Time (h)			0	1	2	3	4
**Average Survival Rate (%)**	β-lactams	Cephalexin	100.00 ± 0.00	91.79 ± 16.12	88.51 ± 3.23	90.60 ± 3.02	89.51 ± 5.35
		Cefradine	100.00 ± 0.00	91.03 ± 2.94	99.39 ± 18.13	95.07 ± 32.93	96.50 ± 10.64
		Cefuroxime	100.00 ± 0.00	96.71 ± 7.91	88.40 ± 1.95	87.73 ± 3.61	89.81 ± 9.40
		Ampicillin	100.00 ± 0.00	104.37 ± 7.17	92.53 ± 11.81	94.22 ± 7.65	90.91 ± 7.88
		Amoxicillin	100.00 ± 0.00	101.38 ± 19.37	100.41 ± 19.84	96.18 ± 8.02	97.86 ± 3.27
	Macrolides	Azithromycin	100.00 ± 0.00	94.20 ± 6.75	91.18 ± 18.18	91.85 ± 27.62	92.42 ± 13.12
		Roxithromycin	100.00 ± 0.00	98.84 ± 8.36	92.77 ± 0.87	94.98 ± 17.11	95.16 ± 6.44
	Aminoglycosides	Gentamicin	100.00 ± 0.00	97.98 ± 21.55	101.61 ± 9.81	98.68 ± 15.26	97.81 ± 19.17
	Tetracyclines	Tetracycline	100.00 ± 0.00	90.96 ± 21.81	93.62 ± 8.75	90.21 ± 5.38	93.87 ± 9.53
		Doxycycline	100.00 ± 0.00	98.48 ± 23.02	91.85 ± 4.87	90.00 ± 18.87	91.36 ± 31.31

SIF = simulated intestinal fluid. Average survival rate at 1 h, 2 h, 3 h, 4 h vs. 0 h; no significance.

## Data Availability

The data that support the findings of this study are available on request from the corresponding author.

## Supplementary Materials

The following supporting information can be downloaded at: https://www.mdpi.com/article/10.3390/microorganisms13061347/s1, Figure S1: Time-kill curve of *Clostridium butyricum* spores in SGF containing β-lactam antibiotics (A) Cefalexin (B) Cefradine (C) Cefuroxime(D) Ampicillin (E) Amoxicillin; macrolides antibiotics (F) Azithromycin(G) Roxithromycin; aminoglycosides antibiotic (H) Gentamicin; tetracyclines antibiotic (I) Tetracycline (J) Doxycycline Data are mean ± SD (n = 3). ns, no significance, * *p* < 0.05, ** *p* < 0.01, *** *p* < 0.001; Figure S2: Time-kill curve of *Clostridium butyricum* spores in SIF containing β-lactam antibiotics (A) Cefalexin (B) Cefradine (C) Cefuroxime (D) Ampicillin (E) Amoxicillin; macrolides antibiotics (F) Azithromycin (G) Roxithromycin; aminoglycosides antibiotic (H) Gentamicin; tetracyclines antibiotic (I) Tetracycline (J) Doxycycline Data are mean ± SD (n = 3). ns, no significance; Table S1: Number of live *Clostridium butyricum* spores in SGF containing antibiotics; Table S2: Number of live *Clostridium butyricum* spores in SIF containing antibiotics.
