# Supplementary material for: Survival and Morphological Changes of Clostridium butyricum Spores Co-Exposed to Antibiotics and Simulated Gastrointestinal Fluids: Implications for Antibiotic Stewardship
Source: Microorganisms. 2025 Jun 10;13(6):1347. doi: 10.3390/microorganisms13061347 (PMC12196182; doi:10.3390/microorganisms13061347)
Supplement: Supplementary file 1 [file microorganisms-13-01347-s001.zip › microorganisms-3597972-supplementary.pdf]

microorganisms
MDPI

Survival and Morphological Changes of *Clostridium butyricum* Spores Co-Exposed to Antibiotics and Simulated Gastrointestinal Fluids: Implications for Antibiotic Stewardship

Yi-Meng Yang[1], Meng-Yue Zhang[1], Ying-Ying Wu[1], Lu Zhang[2] and Yi-Xuan Zhang[1*]

*[1] School of Life Science and Biopharmaceutics, Shenyang Pharmaceutical University, Shenyang, Liaoning 110016, China
[2] Hangzhou Grand Biologic Pharmaceutical INC. Hangzhou, Zhejiang 310030, China
* Correspondence: zhangyxzsh@163.com

Supplementary Materials

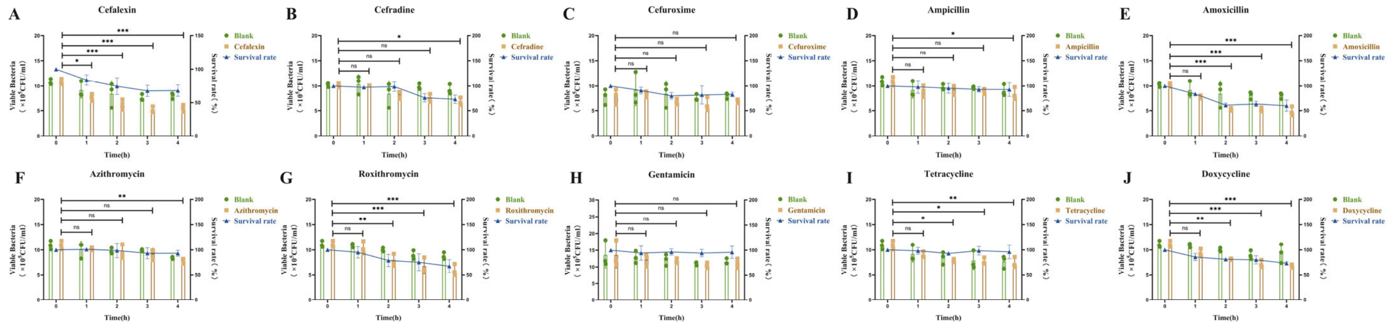

Figure S1. Time-kill curve of *Clostridium Butyricum* spores in SGF containing β-lactam antibiotics (A) Cefalexin (B) Cefradine (C) Cefuroxime(D) Ampicillin (E) Amoxicillin; macrolides antibiotics (F) Azithromycin(G) Roxithromycin; aminoglycosides antibiotic (H) Gentamicin; tetracyclines antibiotic (I) Tetracycline (J) Doxycycline Data are mean ± SD (n=3). ns, no significance,*p < 0.05, **p < 0.01, ***p < 0.001.

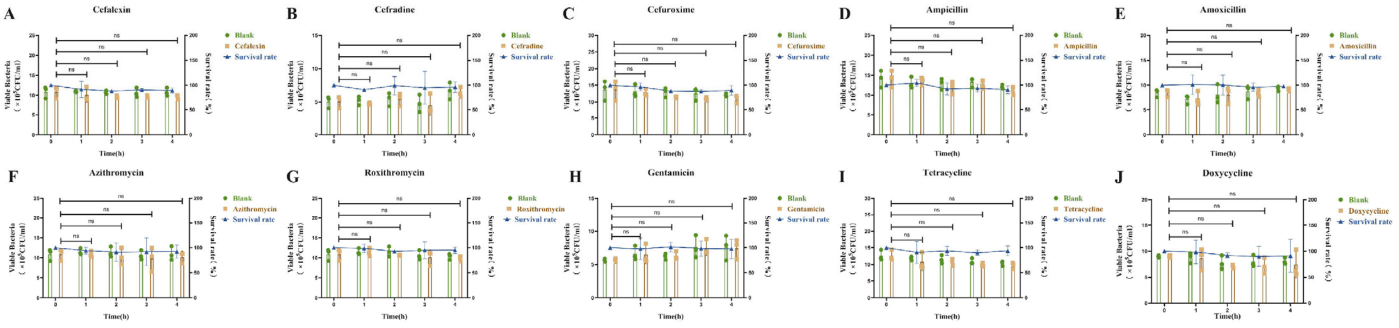

Figure S2. Time-kill curve of *Clostridium Butyricum* spores in SIF containing β-lactam antibiotics (A) Cefalexin (B) Cefradine (C) Cefuroxime(D) Ampicillin (E) Amoxicillin; macrolides antibiotics (F) Azithromycin(G) Roxithromycin; aminoglycosides antibiotic (H) Gentamicin; tetracyclines antibiotic (I) Tetracycline (J) Doxycycline Data are mean ± SD (n=3). ns, no significance.

Table S1. Number of live *Clostridium butyricum* spores in SGF containing antibiotics

Sampling time (h)			0	1	2	3	4
Number of live bacteria(×10^5CFU/ml)	β-lactams	Cephalexin	10.80±0.53	7.70±0.70	6.30±1.05	5.17±0.64	5.50±0.70
		Cefradine	10.27±0.42	9.87±0.40	8.33±0.81	7.53±0.81	6.83±0.78
		Cefuroxime	7.90±1.57	8.40±0.62	6.77±0.57	6.27±1.39	7.07±0.40
		Ampicillin	10.93±0.85	9.00±1.15	9.30±0.96	9.00±0.53	8.40±1.25
		Amoxicillin	10.27±0.42	7.70±0.35	5.20±0.35	5.20±0.44	4.87±0.93
	Macrolides	Azithromycin	10.93±0.85	10.20±0.30	9.60±1.37	9.23±1.07	7.87±0.49
		Roxithromycin	11.10±0.62	10.37±1.22	7.90±1.21	6.77±1.50	5.93±1.14
	Aminoglycosides	Gentamicin	13.57±3.87	12.17±1.83	11.73±0.76	10.43±0.81	11.13±1.40
	Tetracyclines	Tetracycline	10.93±0.85	9.00±0.61	7.83±0.32	7.73±0.67	7.40±1.06
		Doxycycline	11.10±0.62	9.37±0.76	8.13±0.15	7.20±0.72	6.87±0.32

SGF= Simulated Gastric Fluid; The values in this table represent the number of *Clostridium butyricum* spores that survived under the operation of the experimental system.

Table S2 Number of live *Clostridium butyricum* spores in SIF containing antibiotics

Sampling time (h)			0	1	2	3	4
Number of live bacteria(×10⁵CFU/ml)	β-lactams	Cephalexin	10.87±1.46	10.07±1.77	9.50±0.35	9.63±0.32	9.67±0.58
		Cefradine	5.03±0.91	4.73±0.15	5.40±0.98	4.50±1.56	6.43±0.71
		Cefuroxime	13.63±2.93	12.73±1.04	11.43±0.25	10.97±0.45	10.87±1.14
		Ampicillin	14.13±2.11	13.53±0.93	11.97±1.53	12.50±1.01	11.00±0.95
		Amoxicillin	8.47±0.76	7.33±1.40	8.13±1.61	8.40±0.70	9.13±0.31
	Macrolides	Azithromycin	10.87±1.46	10.83±0.78	10.33±2.06	9.77±2.94	10.17±1.44
		Roxithromycin	10.87±1.46	11.37±0.96	10.70±0.10	10.10±1.82	9.83±0.67
	Aminoglycosides	Gentamicin	5.67±0.25	6.47±1.42	6.30±0.61	7.47±1.15	7.43±1.46
	Tetracyclines	Tetracycline	12.60±1.57	10.73±2.57	10.77±1.01	9.83±0.59	9.70±0.98
		Doxycycline	9.00±0.2	8.67±2.03	7.13±0.38	7.20±1.51	7.40±2.54

SIF= Simulated Intestinal Fluid; The values in this table represent the number of Clostridium butyricum spores that survived under the operation of the experimental system.
